# Fast immunosensing technique to detect *Legionella pneumophila* in different natural and anthropogenic environments: comparative and collaborative trials

**DOI:** 10.1186/1471-2180-13-88

**Published:** 2013-04-22

**Authors:** Begoña Bedrina, Sonia Macián, Inmaculada Solís, Roberto Fernández-Lafuente, Eva Baldrich, Guillermo Rodríguez

**Affiliations:** 1 Biótica, Bioquímica Analítica, S.L, Science and Technology Park of Jaume I University, Campus Riu Sec - Espaitec 2, planta baja, Castellón de la Plana, E12071, Spain; 2 Iproma, S.L, Cno.de la Raya 46, Castellón, 12005, Spain; 3 Departamento de Biocatálisis, Instituto de Catálisis y Petroleoquímica, Consejo Superior de Investigaciones Científicas, Campus UAM-CSIC, Cantoblanco, Madrid, 28049, Spain; 4 Institut de Microelectrònica (IMB-CNM, CSIC), Campus Univ. Autònoma de Barcelona, Bellaterra, Barcelona, 08193, Spain

**Keywords:** Legionella, Detection, Immunosensing, Magnetic particles

## Abstract

**Background:**

Legionellosis is an uncommon form of pneumonia. After a clinical encounter, the necessary antibiotic treatment is available if the diagnosis is made early in the illness. Before the clinical encounter, early detection of the main pathogen involved, *Legionella pneumophila*, in hazardous environments is important in preventing infectious levels of this bacterium. In this study a qualitative test based on combined magnetic immunocapture and enzyme-immunoassay for the fast detection of *Legionella pneumophila* in water samples was compared with the standard method, in both comparative and collaborative trials. The test was based on the use of anti-*Legionella pneumophila* antibodies immobilized on magnetic microspheres. The final protocol included concentration by filtration, resuspension and immunomagnetic capture. The whole assay took less than 1 hour to complete.

**Results:**

A comparative trial was performed against the standard culture method (ISO 11731) on both artificially and naturally contaminated water samples, for two matrices: chlorinated tap water and cooling tower water. Performance characteristics of the test used as screening with culture confirmation resulted in sensitivity, specificity, false positive, false negative, and efficiency of 96.6%, 100%, 0%, 3.4%, and 97.8%, respectively. The detection limit at the level under which the false negative rate increases to 50% (LOD50) was 93 colony forming units (CFU) in the volume examined for both tested matrices. The collaborative trial included twelve laboratories. Water samples spiked with certified reference materials were tested. In this study the coincidence level between the two methods was 95.8%.

**Conclusion:**

Results demonstrate the applicability of this immunosensing technique to the rapid, simple, and efficient detection of *Legionella pneumophila* in water samples. This test is not based on microbial growth, so it could be used as a rapid screening technique for the detection of *L. pneumophila* in waters, maintaining the performance of conventional culture for isolation of the pathogen and related studies.

## Background

*Legionella pneumophila* is the major cause of sporadic cases and outbreaks of legionellosis (91.5%), with sero-group 1 being the predominant serotype (84.2%) [1,2], among the 52 species and 70 sero-groups included in the genus *Legionella*[3-5]. Outbreaks of *L. pneumophila* occur throughout the world impacting public health as well as various industrial, tourist, and social activities [6]. Patients with immuno-compromised status are particularly susceptible to this atypical pneumonia [7]. This pathogen is present in both natural [6] and man-made [7] water environments like cooling towers, evaporative condensers, humidifiers, potable water systems, decorative fountains and wastewater systems (risk facilities). Human infection can occur by inhalation of contaminated aerosols [8]. Colonization at human-made water systems has been associated with biofilms yielding only some free bacterial cells [1,9,10]. Moreover, rapid fluctuations of the concentration of *L. pneumophila* at risk facilities have been reported [11], as well as persistence of *L. pneumophila* in drinking water biofilms mostly in a viable but non-culturable state (VBNC) [12], which has also been confirmed even after treatments with chlorine used to disinfect cooling towers [13,14]. In fact, *L. pneumophila* becomes non-culturable in biofilms in doses of 1 mg/L of monochloramine, making culture detection of this pathogen ineffective [15]. The effectiveness of treatments on *Legionella pneumophila* (chlorine, heat, ozone, UV, monochloramine) has been mainly evaluated based simply on cultivability and that could not be a real indicative of the absence of intact viable cells [16-18].

Official methods for *Legionella* detection are based on the growth of the microorganism in selective media [19,20]. At least 7 to 15 days are required for obtaining results due to the slow growth rate of the bacterium. Culture detection also shows low sensitivity, loss of viability of bacteria after collection, difficulty in isolating *Legionella* in samples contaminated with other microbial and the inability to detect VBNC bacteria [21]. Therefore, the development of a rapid and specific detection method for *L. pneumophila* monitoring and in real time would be crucial for the efficient prevention of legionellosis. Polymerase chain reaction **(**PCR) methods have been described as useful tools for *L. pneumophila* detection [22,23]. PCR reportedly provides high specificity, sensitivity, and speed, low detection limits and the possibility to quantify the concentration of the microorganisms in the samples using real-time PCR. However, it requires sophisticated and expensive equipment, appropriate installations and trained personnel [24]. PCR inhibiting compounds present in environmental samples may cause false negatives. Inhibition control is strongly recommended in those cases. Samples having inhibition must be diluted and retested. False positives can be caused by the inability of PCR to differentiate between cells and free DNA [25]. Finally, the cell number assigned to a certain amount of target genes varies by one order of magnitude depending on the growth phase and bacteria species, limiting the capability of PCR test for accurate bacterial quantification [26]. Immuno-detection has provided the basis for the development of powerful analytical tools for a wide range of targets. During the last years, the number of publications in this field has increased significantly [27]. Traditionally, the most common method applied to microorganism detection has been the enzyme-linked immunosorbent assay (ELISA). The main drawback of ELISA is the high detection limit generated; which is often between 10^5^ and 10^6^ CFU/mL [28]. This limit may be improved to 10^3^ and 10^4^ cells/mL using more sensitive detection methods [29,30]. The immobilization of antibodies onto the surface of magnetic beads to obtain immunomagnetic beads (IMB) has promoted the development of immunomagnetic separation (IMS). Thereby, IMS provides a simple but powerful method for specific capture, recovery and concentration of the desired microorganism from heterogeneous bacterial suspension [23,31-34].

A test based on IMS by anti-*L. pneumophila* immuno-modified magnetic beads (LPMB), coupled to enzyme-linked colorimetric detection has been proposed for the rapid detection of *L. pneumophila* cells in water samples [35]. In this study, intensive comparison of this immunomagnetic method (IMM) with the culture method is presented.

## Results

### Comparative trial with natural samples

The IMM test was applicable to detection of *L. pneumophila* in water samples. A total of 459 water samples, comprising both naturally contaminated and artificially contaminated samples were examined for the presence of *L. pneumophila* using the reference culture method (ISO 11731-Part 1) and the IMM test in parallel. The parameters for this comparison study were calculated from the results summarized in Table 1 as it is described in the Methods section. Sensitivity and specificity were estimated as 96.6% (284/294) and 88% (145/165), respectively for the IMM. This means that a proportion of actual positives and negatives are correctly assigned by the IMM test. False positives and false negatives were estimated as, respectively, 12.0% (20/304) and 3.4% (10/294). Some “false” positives could be related to problems in the culture method, as stated in the background that presents some limitations under different circumstances [12,15,21]. In fact, the PCR analysis of some of the samples initially considered false positives confirmed later the existence of DNA from *L. pneumophila* in those samples (results not shown), suggesting a failure of the culture method. From the point of view of the IMM as a screening test with culture confirmation, presumptive test negative results can be added to the true negatives. In this case sensitivity and specificity were estimated as, respectively, 96.6% (284/294) and 100% (0/165) for the IMM. False positives and false negatives were estimated as, respectively, 0% (0/324) and 3.4% (10/294). The low false negative ratio suggests that the IMM is very reliable.

**Table 1:** Comparison of the immunomagnetic method with the standard culture method

	**Immunomagnetic method (IMM) as screening assay**			
	**Without confirmation **^**a**^		**With confirmation **^**b**^	
Culture method (ISO 11731)	+	―	+	―
+	284	10	284	10
―	20	145	0	165

^a^ Presumptive IMM results.

^b^ Confirmed IMM results.

Efficiency of the IMM as screening assay without confirmation was estimated as 93.5% (429/459). The IMM with confirming culture method had an efficiency of 97.8%. This means that results obtained with the IMM test exhibited a high agreement with the reference culture method.

### Detection limit

The detection limit of the IMM test was determined by testing water samples spiked with different *L. pneumophila* (ATCC 33152) concentrations at 5 different levels (Table 2). The detection limit was defined as the lowest number of cultivable *L. pneumophila* organisms (confirmed by culture) that can be detected with a probability of 50%. On the basis of this criterion, the detection limit of IMM for *L. pneumophila* was determined as 93 CFU per volume examined for the studied matrices. Here the volume examined is the filtered volume of the original water sample.

**Table 2:** **Summary of immunomagnetic test and ISO reference method results for the estimation of LOD**_**50**_

**Level no.**	**Culture count, CFU/mL**	**IMM presumptive positive/total portions tested**
1	0	0/6
2	3.4	0/10
3	15.1	14/30
4	20.4	7/10
5	68.3	10/10

### Collaborative trial

Table 3 shows the results of the eleven accepted laboratories that have evaluated the IMM test. The concentrations estimated by the color chart of the IMM test were highly coincident with the reported culture results for each one of the three groups of samples prepared with certified reference material (pills) containing *L. pneumophila*. For the two pills used as negative control, not having *L. pneumophila*, this bacterium was not detected by any of the two methods (culture isolation and IMM test) in any of the participating laboratories. Coincidence between both methods was of 95.8%. Comparison gave good results, with clear coincidence with the standard culture method but a higher rate of analysis.

**Table 3:** ***Legionella pneumophila *determination in collaborative trial, Log (CFU/9 mL) (by participant no.)**^**a**^

		**Culture results**											**Immunomagnetic results**																					
**Level of spiking**^**b **^**Log**_**10 **_**CFU/9 mL**	**Pill**	**Culture count log**_**10 **_**CFU/9 mL**^**c**^											**Estimated magnitude order log**_**10 **_**CFU/9 mL**											**Qualitative results**^**d**^										
		1	2	3	4	5	6	7	8	9	10	11	1	2	3	4	5	6	7	8	9	10	11	1	2	3	4	5	6	7	8	9	10	11
0	P_6_	ND	ND	ND	ND	ND	ND	ND	ND	ND	ND	ND	ND	ND	ND	ND	ND	ND	ND	ND	ND	ND	ND	A	A	A	A	A	A	A	A	A	A	A
	P_8_	ND	ND	ND	ND	ND	ND	ND	ND	ND	ND	ND	ND	ND	ND	ND	ND	ND	ND	ND	ND	ND	ND	A	A	A	A	A	A	A	A	A	A	A
2.23	P_4_	2.83	2.22	2.21	2.47	2.57	2.11	2.38	2.23	2.73	1.98	2.32	3.0	<3.0	3.0	<3.0	<3.0	<3.0	2.0	2.0	3.0	2.0	3.0	P	P	P	P	P	P	P	P	P	P	P
	P_7_	2.11	2.16	2.36	2.25	2.13	2.11	2.10	2.01	2.17	1.90	2.32	<4.0	<3.0	<4.0	<4.0	<3.0	3.0	3.0	2.0	<4.0	2.0	3.0	P	P	P	P	P	P	P	P	P	P	P
2.88	P_1_	3.07	2.86	3.12	3.19	3.04	1.99	2.99	2.96	2.69	2.78	2.85	4.0	3.0	3.0	<4.0	3.0	3.0	3.0	3.0	3.0	3.0	3.0	P	P	P	P	P	P	P	P	P	P	P
	P_3_	3.13	2.90	3.11	3.13	3.07	2.29	2.61	2.51	2.77	2.57	2.77	3.0	3.0	3.0	<4.0	3.0	2.0	3.0	2.0	<4.0	3.0	3.0	P	P	P	P	P	P	P	P	P	P	P
3.82	P_2_	4.01	4.23	4.03	3.93	3.76	3.59	3.49	3.56	3.21	3.59	4.22	4.0	>4.0	>4.0	<4.0	4.0	>4.0	4.0	3.0	4.0	>4.0	4.0	P	P	P	P	P	P	P	P	P	P	P
	P_5_	3.92	4.20	4.30	3.64	3.63	3.94	3.77	3.66	3.97	3.61	3.95	4.0	4.0	4.0	4.0	4.0	>4.0	4.0	3.0	4.0	>4.0	>4.0	P	P	P	P	P	P	P	P	P	P	P
	P_9_	3.33	4.20	3.91	3.89	3.92	3.71	3.48	3.63	3.97	2.91	3.99	4.0	4.0	4.0	4.0	4.0	>4.0	>4.0	>4.0	3.0	4.0	4.0	P	P	P	P	P	P	P	P	P	P	P

^a^ This table includes only results from participating laboratories that were not excluded due to obvious deviation from the trial protocol.

^b^ Concentrations calculated from the results provided by the 11 participating laboratories, assigned to the used reference materials (pills).

^c^ ND, not detected.

^d^ A, absence; P, presence.

## Discussion

This study confirms the suitability of the IMM test for the detection of *L. pneumophila* in water samples. The final protocol comprised sample pre-concentration by filtration and resuspension, magnetic capture using immunoactivated beads, and colorimetric enzyme-linked immunodetection in just 1 h of analysis, while the standard protocol requires 7–14 days. Sensitivity (96.6%), specificity (100%), false positives (0%), false negatives (3.4%), and efficiency (97.8%) were determined. The LOD50 was only 93 CFU of *L. pneumophila* in the volume examined for the selected matrices, which is significantly below the values reported for other conventional methods such as ELISA. This occurs even though some of the samples (mainly from cooling towers) presented viscosity and dirtiness that made handling difficult.

## Conclusions

In view of these results, the IMM test could be a valuable tool for the rapid, simple and robust detection of free *L. pneumophila* at risk installations, in a weekly and even daily basis, contributing to minimize the risk of outbreaks by this pathogen. At theses environments, presence of *L. pneumophila* or a high percentage of positive points, have been identified as factors contributing to explain case onset [36]. The reported combination of magnetic capture and enzyme-immunoassay provides a user-friendly and extremely easy to use assay format, which is a valuable low-cost tool for the implementation of in situ surveillance, development of Water Safety Plans, or fast screening of water samples. In combination with other established techniques, such culture and PCR, addressed to isolation and identification of *L. pneumophila*, IMM could be useful for an integral surveillance. From the results presented in this study, Legipid IMM test is a very promising tool to fight against legionellosis and similar configurations could be used to detect other dangerous pathogens.

## Methods

### Comparative trial

Intensively water testing was made to compare the IMM to the ISO 11731 reference culture method. Tap, cooling tower and natural water samples were collected from different locations and different seasons during three years in the period 2008–2011. A total number of 459 water samples were tested. From these samples, 189 were naturally contaminated samples and 270 were artificially contaminated samples. Distribution of naturally contaminated samples was the following: 84 samples from cooling towers, 94 samples from tap water, 8 samples from water wells and 3 waste water samples. Distribution of artificially contaminated samples was the following: 104 samples from cooling towers, 166 samples from tap water. Both the collection *L. pneumophila* strain (ATCC 33152) and an environmental isolate of *L. pneumophila* sg 1 were used as inoculums to prepare artificially contaminated samples. *Legionella pneumophila* was grown for 3 days on BCYE agar (Buffered Charcoal Yeast Extract) supplemented with glycine, vancomycin, polymixine and cycloheximide (GVPC medium) to obtain exponential-phase cultures. These cultures were used to inoculate water samples. Each sample was tested for the level of background flora by standard plate count of dilutions series of each type of sample. The concentration of *Legionella pneumophila* ranged from 10^2^ CFU to 10^7^ CFU in the volume examined, between 0.1 L to 1.0 L (usually 1.0 L). Generally, the level of total bacterial counting was below 50 CFU/mL for the tap water samples, and this level was ranging from 10^2^ to 10^5^ CFU/mL for cooling tower water samples, most of them between 10^3^ and 10^4^ CFU/mL. Each of these examined volumes were concentrated by filtration through 0.4-μm-pore-size, 47-mm-diameter polycarbonate sterile membranes (Sartorius, Germany), following the instructions of the International Standard method ISO11731-Part 1. After filtration, each membrane was directly placed in a screw cap sterile container containing 10 mL of the reagent L0 (Biótica, Spain). Then *L. pneumophila* was eluted by vortex mixing for 2 min. An average of 47% of the seeded *L. pneumophila* organisms were recovered by filtration. This concentrate represented the prepared sample. The volume of this sample was divided into two portions: 9 mL for IMM test and 1 mL for the culture test. The positivity or negativity of the water samples by the IMM was visually recorded by the colorimetric end-point reaction.

### Detection limit

The detection limit was determined considering validation protocols of international certification bodies [37,38]. Both tap and cooling tower waters were collected and tested negative for the *L. pneumophila* before its use as matrices. *Legionella pneumophila sg 1* (ATCC 33152, Laboratoire BioRéférence, ipl-Groupe, France) was resuspended into 20 mL of a sterile saline solution at room temperature under gently agitation. These 20 mL-suspensions were used to inoculate one liter of selected matrices. Five levels of target contamination were prepared to obtain fractional positive results by the IMM method. Level one corresponded to absence of the target organism, level two corresponded to a proportion of IMM positive results minor than 50%, level three corresponded to a proportion of IMM positive results around the 50%, level four corresponded to a proportion of IMM positive results higher than 50%, and finally level five corresponded to a proportion of IMM positive results of 100%.

These artificially contaminated 1.0 L-samples left to equilibrate for 15–16 hours at 4°C prior starting analysis, to stabilize the inoculated target organism. Each 1.0 L-sample was then divided into ten 100 mL-aliquots as replicates. A total of 66 100 mL-aliquots were examined. Each of these 100 mL-aliquots was concentrated by filtration following the instructions of the International Standard Method ISO11731-Part 1. The volume of each 10 mL-concentrated sample was divided into two portions: 9 mL for IMM test and 1 mL for the culture test. The positivity or negativity of the water samples by the IMM was visually recorded by the colorimetric end-point reaction. The proportion of positive results by the IMM was determined for each batch of ten 100 mL-replicates for each sample.

### Reference culture method

For water testing and detection limit study, ISO11731-Part 1 was applied. Water samples were concentrated as described above. Briefly, after filtration of the volume examined, 0.1 mL-portion of the prepared sample was spread on the surface of BCYE agar (Buffered Charcoal Yeast Extract) medium supplemented with glycine, vancomycin, polymixine and cicloheximide (GVPC medium) (bioMérieux, Spain), while a 9 mL-portion of the prepared sample was tested by the IMM. The samples inoculated with high concentrations of *L. pneumophila* were first diluted with the same water matrix to ensure the count of colony forming units (CFU). The cultures were incubated for 10 days at 37± 1°C in humid atmosphere containing 5% of CO_2_.

### Immunomagnetic technique

The IMM test (Legipid® *Legionella* Fast Detection kit, Biótica, Spain), contained different reagents (L0, L1, L2, L3, L4, L5, and L6) and an easy to handle magnetic particle concentrator comprised by a magnet and two glass cuvettes. Unless otherwise stated, aall steps were conducted at room temperature in the magnetic particle concentrator. Nine milliliters portions of each prepared sample for water testing and detection limit studies were transferred to the kit glass cuvette, and 1 mL of L1 reagent containing *Legionella pneumophila*-binding magnetic beads (LPBM) suspension was added. The mixture was mildly rocked for 15 minutes. LPBM separation was performed by applying a magnet to the cuvette for 5 minutes, and the supernatant was discarded overturning the cuvettes. The LPBM was resuspended/washed with 5 ml of reagent L2 followed by magnetic separation as above. The LPBM were then incubated in 1 ml of reagent L3 for 10 minutes, were captured with the magnet (3 min), was resuspended/washed three times with 5 ml of reagent L2, and were magnetically captured again (3 min). Reagent L4 includes two powder co-substrates (1.3 mg each one) for the colorimetric reaction and it was dissolved in 1.3 mL of reagent L5. The LPBM were resuspended in this solution under gentle agitation for 2 minutes to generate the signal. Then 100 μL of L6 reagent was added to stop the reaction. The mixture was rocked for 1 minute. The LPBM were captured again as described above, and after 5 minutes, the color was compared with a negative control (without *L. pneumophila*). The kit is intended to provide a semi-quantitative measure of *L. pneumopila* concentration, by interpolation of the color developed by the tested sample in the supplied color chart. If the colorimetric reaction showed no difference between sample and negative control after two minutes, then the reaction was allowed to proceed for 10 minutes before stopping to trap low positives which correspond to an estimate level around the LOD50 of the IMM test.

A test is considered positive if at 2 minutes or before 10 minutes color difference appears with the control. A positive *L. pneumophila* test must have a color higher than the color control at 2 minutes from starting colorimetric reaction. Then reaction was stopped following the protocol instructions. General estimation of the level of *L. pneumophila* in the sample was obtained comparing the test color with the color chart. If there was no color difference at 2 minutes, the reaction was allowed continue up to 10 minutes and then it was stopped. A positive *L. pneumophila* test must have a color higher than the color control at 10 minutes from starting colorimetric reaction. In this case, the estimated level of *L. pneumophila* was low, up to two orders of magnitude (10^2^ CFU/volume examined). A negative *L. pneumophila* test was considered if there was no color difference with the control after 10 minutes.

### Calculation of performance characteristics

The test performance characteristics (specificity, sensitivity, false positives, false negatives, and efficiency) of the IMM were determined. Available ISO guides are designed to validate methods based on the microbial growth and the key issue is the “growth unit” capable to growth in a nutrient media. Although the qualitative IMM kit is not based on the growth unit, a first categorization of the presumptive results was obtained by using a two-by-two contingency table, following the scheme provided by the norm ISO/TR13843 [39]. IMM presumptive results were compared with the ones obtained with the reference method (ISO11731). These results were divided into four categories: (a) number of presumptive positives by the IMM found positive by the reference culture method (true positives), (b) number of presumptive negatives by the IMM found positive by the reference culture method (false negatives), (c) number of presumptive positives by the IMM found negative by the reference culture method (false positives), and (d) number of presumptive negatives by the IMM found negative by the reference culture method (true negatives). The sensitivity was defined as the ability of IMM to detect the target microorganism compared to the reference culture method, as follows: (a × 100) / (a + b). The specificity is defined as the inability of the IMM to detect the target microorganism when it is not detected by the reference culture method, as follows: (d × 100) / (c + d). Finally, the efficiency is a general single parameter, which gives the agreement between the response obtained by the IMM and the reference culture method, as follows: (a + d) × 100 / n, where n is the total number of tests. The percentage of false positives is calculated as (c × 100) / (a + c), and the percentage of false negatives is calculated as (b × 100) / (b + d).

A qualitative test can be used as screening assay with confirmation. Only in this case, positive presumptive result confirmed as negative by the confirming culture method can be re-categorized as true negative. Performance characteristics were also calculated with this consideration, according to the guidelines of certification bodies [40].

### Calculation of detection limit

Detection limit was established as the lowest number of cultivable *L. pneumophila* organisms that can be detected with a probability of 50%. This parameter so-called LOD_50_ is estimated using a statistical model (Spearman-Kärber test) but not directly measured [37,38,40].

### Collaborative trial

A collaborative trial involving twelve independent laboratories was performed to evaluate the validity of the IMM by testing identical samples. The collaborative trial was designed and conducted according to internationally accepted guidelines [37,41-49]. It has been shown that concentration methods can have highly variable recovery rates, making difficult to obtain identical samples especially for low concentrations of *L. pneumophila*[50]. Since the objective was the evaluation of the detection part of the IMM, the tested sample simulated the concentrated sample that is habitually obtained in the laboratory from an original sample, thus avoiding the concentration phase.

In this collaborative trial, a microbiological reference material in pill format was used (BaCuanti, Labaqua, Spain). According to the manufacturer´s instructions, water samples were obtained by diluting these pills. The twelve participating laboratories received pills of *L. pneumophila* at four levels: (i) pills P_6_ and P_8_ as negative control, (ii) pills P_1_ and P_3_, containing a medium level of *L. pneumophila*, (iii) pills P_2_, P_5_ and P_9_, containing a high level of *L.pneumophila*, and (iv) pills P_4_ and P_7_, containing a low level of *L. pneumophila*.

To minimize any interlaboratory variability, all the required reagents were purchased from Biótica, Bioquímica Analítica S. L. Each participant received a detailed protocol describing the culture technique, the immunomagnetic run, and a reporting form to record the obtained results.

### Samples preparation

The pills were supplied to the participating laboratories into individual sealed vials. For sample reconstitution, the safety seal was removed under aseptic conditions, the vial was opened, and 20 mL sterile distilled water were added, allowing to mix for 10 min at room temperature, gently shaking every 2 min. Sterile water was added up to a final volume of 100 mL. Then, three serial decimal dilutions (10^-1^, 10^-2^, and 10^-3^) of each sample were prepared.

### Reference culture method

Determination of *L. pneumophila* by culture isolation was conducted in accordance with the ISO 11731-Part 2. Five milliliters of each sample, as well as its corresponding 10-fold serial dilutions were filtered through cellulose ester membranes (11406-47-ACN; Sartorius, Germany). The membranes were placed on the surface of the BCYE-α+GVPC medium (bioMérieux; Spain) and were incubated at 37°C, preferably in a 5% CO2 atmosphere for a period between 5 and 10 days.

### Immunomagnetic technique

Analysis using the IMM test kit was performed in accordance to the protocol described previously. Results were reported as presence/absence in 9 mL, and the aproximate concentrations of *L. pneumophila* were estimated by intercalation of the end-point colour developed in the analysed sample in the colour chart provided by the manufacturer. Accordingly, samples similar to the negative control one were labelled as <LOD (limit of detection), colour between the negative control and the first colour mark corresponded to 10^2^–10^3^ CFU/9 mL, colour similar to the first colour mark corresponded to 10^3^ CFU/9 mL, colour between first and the second colour mark corresponded to 10^3^–10^4^ CFU/9 mL, colour similar to the second colour mark corresponded to 10^4^ CFU/9 mL, and colour darker than the second colour mark was indicative of >10^4^ CFU/9 mL.

### Statistical data analysis

The results reported by eleven of the twelve participating laboratories were evaluated following statistical methods described in the ISO/DIS 13528. One laboratory was rejected due to incorrect application of the trial protocol.

## Competing interests

Financial competing interests: GR and BB are employed at Biótica from which test for *Legionella* detection was supplied. The author(s) declare that there are no competing interests. Non-financial competing interests: The authors declare that there are no non-financial competing interests.

## Authors’ contributions

GR and RF conceived the study. IS, BB, GR designed the experiments. RF and GR wrote the paper. IS, BB, SM performed experiments and analyzed data. RF and EB helped with research design. IS, SM, RF, GR helped with manuscript discussion. IS provided samples. RF, EB helped to draft the manuscript. All authors have read and approved the final manuscript.
